# Advancing preclinical models of psychiatric disorders with human brain organoid cultures

**DOI:** 10.1038/s41380-022-01708-2

**Published:** 2022-08-10

**Authors:** Thomas Anthony Dixon, Alysson R. Muotri

**Affiliations:** 1 Department of Psychiatry, University of California San Diego, La Jolla, CA 92093 USA; 2 Department of Pediatrics and Department of Cellular & Molecular Medicine, University of California San Diego, School of Medicine, Center for Academic Research and Training in Anthropogeny (CARTA), Kavli Institute for Brain and Mind, Archealization Center (ArchC), La Jolla, CA 92037 USA

**Keywords:** Stem cells, Schizophrenia

## Abstract

Psychiatric disorders are often distinguished from neurological disorders in that the former do not have characteristic lesions or findings from cerebrospinal fluid, electroencephalograms (EEGs), or brain imaging, and furthermore do not have commonly recognized convergent mechanisms. Psychiatric disorders commonly involve clinical diagnosis of phenotypic behavioral disturbances of mood and psychosis, often with a poorly understood contribution of environmental factors. As such, psychiatric disease has been challenging to model preclinically for mechanistic understanding and pharmaceutical development. This review compares commonly used animal paradigms of preclinical testing with evolving techniques of induced pluripotent cell culture with a focus on emerging three-dimensional models. Advances in complexity of 3D cultures, recapitulating electrical activity in utero, and disease modeling of psychosis, mood, and environmentally induced disorders are reviewed. Insights from these rapidly expanding technologies are discussed as they pertain to the utility of human organoid and other models in finding novel research directions, validating pharmaceutical action, and recapitulating human disease.

## Introduction

Psychiatric diseases have been notoriously difficult to study from a basic science perspective, and unfortunately, consolidated mechanisms of mood, psychotic, and environmentally induced disorders at the most fundamental level have eluded researchers for centuries. These obstacles to discovery come from both technical and ethical perspectives. From a technical viewpoint, understanding psychiatric disorders relative to neurological diseases is difficult as brain ‘lesions,’ or signals in cerebrospinal fluid (CSF), electrophysiological, or imaging studies are not as robust and apparent in disorders we consider psychiatric in nature. Ethically speaking, preclinical experimentation of in vivo and in vitro central nervous systems (CNSs) involves unique sets of moral implications, briefly discussed below.

It is worth noting that several disorders with known genotype lead to cognitive and behavioral phenotypes that may fall under the umbrella of psychiatry. In this review we will focus primarily on neurodevelopmental and acquired psychiatric disorders without a definitive genetic cause, as these present a particularly distinct and under-reviewed challenge in creating preclinical models.

## Ethical considerations

**Fig. 1:**
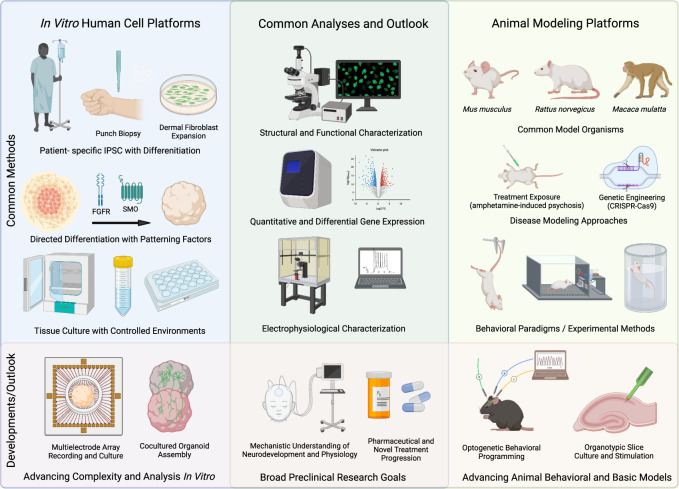
Comparison of in vitro and animal modelling platforms for preclinical research. Schematic illustrating commonalities and differences between preclinical research platforms of in vitro human cell platforms (left, blue) and animal modeling (right, yellow), with shared analyses (center, green). Ongoing developments in human cell platforms (bottom left), animal modelling (bottom right), and shared research goals (bottom center). Individual boxes described from left to right, all content generalized and streamlined for ease of depiction. (Left) Human cell-based platforms use punch biopsies from patients and controls to derive dermal fibroblasts, then differentiated in directed methods to specified 3D organoids, which can be cultured in standardized and controlled tissue culture. Developments including multi-electrode array recording and cocultured fused organoids (‘assembloids’) are among the more recent advances in the field toward increased complexity of culture and analysis (bottom left). Human cell based and animal modeling platforms use several shared analytic techniques for similar readouts (center), and have similar goals of understanding neurodevelopment mechanistically, as well as novel treatment development (bottom center). Animal models use common model species which can be induced to disease states with specific treatments or genetic programming (right). Behavioral tests are used as experimental methods for phenotypic readouts, and optogenetics and other tissue based methods represent newer developments in the use of animal models for preclinical and basic research (bottom right). Schematic created with BioRender.com.

The ethical challenges in studying psychiatric disorders preclinically lie in several realms and are not solved by the review of advanced culture methods described herein. The chief modalities currently involved in preclinical testing involve testing on animals, typically primates and rodents, with burgeoning contributions from in vitro human cell platforms (Fig. 1). Experimenting on living humans to determine developmental paradigms has a historically complex record. However, since voluntary consent was mandated by the Nuremberg Code of 1947 [1], the developing brain has not been subject to invasive research to determine basic neurobiological mechanisms.

While we use animals as surrogates for testing disorders of higher-order cognition, consciousness, and behavior, as animals are unable to provide consent, one must use a principle of comparative beneficence to justify animal testing. As animals display complex behaviors that necessitate some degree of higher-order thinking and cognition that is generally considered to be related to cerebral complexity, one may also expect a degree of subjective suffering in research animals whose experience likely resides somewhere on a continuum of consciousness. Researchers already assume a degree of consciousness with ongoing and historical experiments, as an example rodents have been used as surrogates for testing in consciousness studies, including with thalamic lesions [2]. Other animals outside the order Rodentia have complex behaviors that may correlate to consciousness to a varying degree. Macaque monkeys demonstrate signaling when they do not see a stimulus. Birds, fish, cephalopods, and even insects are capable of sophisticated, learned, non-stereotyped behaviors which have been associated with consciousness in humans [3]. Simply put if we are using animals in consciousness studies, we must also describe them with the capacity for equivalent degrees of suffering and pain, leading to an ethical quandary in their use for the study of diseases thought to be human-specific.

The ethics landscape of human organoid research is an emerging topic beyond the scope of the present article, and while several viewpoints have been published in recent years, there exists no consensus framework on best practices or broadly adopted research restrictions [4–9]. A recent “decision-tree” has been proposed as a roadmap for researchers working with complex human brain organoids that may reach some level of consciousness [10]. Another preliminary principle is that human subjects should be consented to all intended use of derived organoids, especially noting use in studies of complex oscillatory activity [11]. Further ongoing questions involve privacy and health data concerns with patient-derived cultures, concerns regarding commercialization, patenting, and biobanking of patient-derived samples, as well as efforts to minimize or prevent potential pain and suffering resulting from de novo manifestation of subjective experience or awareness [10].

### Development and comparison of in vitro neuronal cultures

An alternative or perhaps a complement to preclinical animal testing that has been emerging for greater than a century is the study of neuronal cultures in controlled laboratory settings. The cultures were studied initially in explant tissue surviving ex vivo, and this field has now developed into sophisticated analyses of complex human stem cell-based neural networks growing in three dimensions (3D). At the present relatively advanced stage, these networks have limited ability for efferent output and cannot yet display phenotypic behavior, a hallmark of psychiatric disease. However, significant advancements in cell culture techniques toward surrogacy for in vivo human neurodevelopment warrant discussion as an alternative validated preclinical complement to the paradigm of animal models.

Beyond preclinical testing modalities, most aspects of psychiatric illness, including etiologies, remain controversial. Mechanistic origins described from polygenic to environmental contributions as well as neurodevelopmental alterations in utero through adolescence and adult life have all been suggested and researched extensively. The myriad of potential etiologies may be investigated by several means, and in the case of commonly cited and complex polygenic origin, it has been argued that 3D cell models would have utility in decoding the impact of genetic variants on pathological brain development leading to mental illness [12].

Moving forward in this review, we will begin with a comparison of animal models for psychiatric research, followed by an overview of the development of advanced in vitro cell cultures, and then succeeded by subsections of relevant psychiatric models. This work builds on prior reviews of cell culture technology, including modeling of the neurotypical CNS and its development with its unique set of complexities, described extensively in prior work [13–21] and in specific disease models and discussion predominantly of 2D iPSC systems [22–30].

#### Brief comparative analysis for rodent models in human psychiatric research

As animal models, in particular rodent models, have been used extensively in a preclinical context to characterize genetic and neural mechanisms of psychiatric disorders and to facilitate new treatments [31], a limited, focused review follows in this section, providing a background for further advancements in preclinical models with in vitro techniques. Diverse animal models of psychiatric illness have been developing for decades, markedly as the ‘learned helplessness’ model with Beagles, published in 1972 [32], and with the forced-swim test [33] and tail suspension test [34] in mice published in the following decade. A full-scale review of further development in animal models for psychiatric diseases until the present is beyond the scope of this review, and we will instead focus on relevant comparisons between animal models and advancing in vitro models (Fig. 1). Starting with psychosis-like disease, murine models of psychosis have been extensively reviewed elsewhere [35]. Briefly, several modalities are used for psychosis induction with varying degrees of human relevance, including: treatment with stimulants, dopamine agonists, N-methyl-D-aspartate (NMDA) receptor antagonists, genetic manipulations of dysbindin1, disrupted in schizophrenia 1 (DISC1) mutants, G-protein subunit (Gsα), Catechol-O-methyltransferase, and environmental manipulations including prenatal exposure to methylazoxymethanol acetate (MAM), a cell division inhibitor, and non-pharmacological interventions including isolated rearing [36] and prolonged use of restraints [37].

Recent advances in murine models have correlated hallucination-like precepts in mice, through ketamine administration, and optogenetically boosting striatum dopamine signaling, which responded in an expected way to haloperidol administration [38]. As noted there is an ‘inability to truly interpret the motivation or meaning of the vast majority of animal behaviors.’ Behavioral tests with read-outs of ‘behavioral despair’ have been described as ‘anthropomorphic leap,’ which is argued in many cases to not be convincingly related to pathophysiology (considered the anthropomorphic fallacy) [39]. Other techniques for psychiatric modeling include high-frequency headshakes used for assaying psychedelic compounds [40], increased immobility while suspended by tails, decreased sucrose preference, and decreased maze exploration used for depression and anxiety (Fig. 1)[41]. Overall, animal models have diverse applications and have the unique advantage of displaying phenotypic behavior in a living system. For example, as attention deficit hyperactivity disorder is primarily diagnosed with behavioral disturbance, the animal model of spontaneous hypertensive rats has been invaluable as relevant behaviors are modeled, including behavioral variability, deficient response re-engagement, increased error rates, and most pertinently impaired sustained attention and hyperactivity not present in novel, non-threatening conditions but developing over time with infrequent reinforcers [42]. A further example involves rat models of autism spectrum disorder where Fragile X messenger Ribonucleoprotein 1 (Fmr1) knock out rats pups spend less time playing with fewer ultrasonic communications, as well as other repetitive behaviors [43].

Currently, available rodent animal models are used frequently for basic drug screening and investigations in neuroscience, however they are still time-consuming, costly, and do not study the molecular and cellular effects of these drugs on the human brain [44]. While there has been diverse development of several different types of modeling with animals, many divergences widening the gap between animal modeling and human disease are worthy of discussion.

Focusing first on murine models concerning inflammation, recent analysis has found that murine model transcriptional responses to inflammation correlate poorly with humans, particularly found when comparing murine models of trauma, burns, and endotoxemia, which did not correlate with highly consistent genomic response to these insults in human patients [45]. This serves a useful representation of a pitfall of an animal preclinical model, particularly as inflammation remains a frequently deliberated topic in psychiatric etiologies, with notably uncertain causal direction, hypothesized to have feedback bidirectionally between inflammation and neuropsychiatric manifestations [46]. While a full discussion of the interplay between the immune system and neuropsychiatry is beyond the scope of this review, for the purposes of discussion of psychiatric preclinical models, it is worth noting that several species-difference factors, including human and mouse divergence between 65 and 96 million years ago, difference in size and lifespan, as well as evolution in different ecological niches contribute to ‘significant differences between mice and humans in immune system development, actuation, and response to challenge in innate and adaptive arms’ [47].

Further comparing species, rodents are social creatures with some aspects that humans would find familiar including forms of auditory communication (with ultrasonic squeaks) relating to purported positive/negative affect [48]. However it should be clear to all observers that the up to 96 million years [49] that we have diverged in evolution from mice has led to striking species differences that would be certain to limit comparative impact. Furthermore, psychiatric disease has complex behavioral phenotypes, which as far as we know, are specific to humans.

**Fig. 2:**
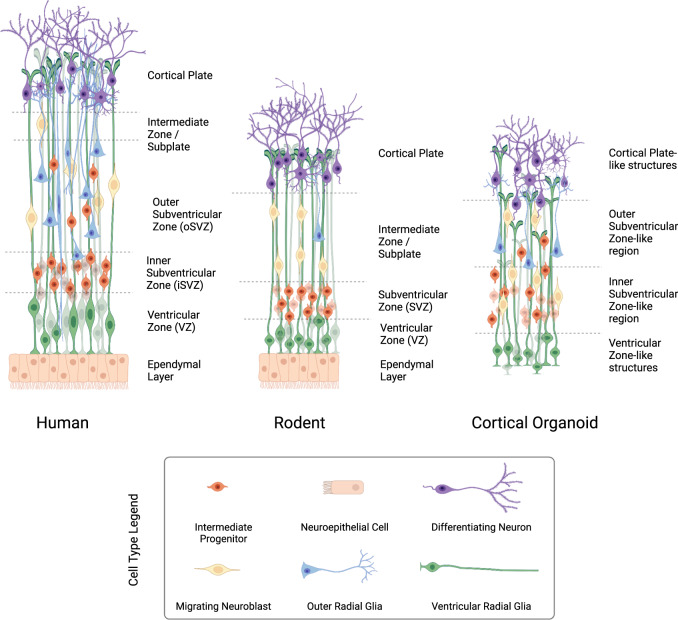
Comparison of neural layers found in development of the mammalian cerebral cortex. Simplified illustration of human, rodent, and organoid model systems expanding cortical neural layers, with cell types distinguished by color and morphology with legend on left. The human neural layers (left) are marked by an expanded subventricular zone with a unique outer layer, named the outer subventricular zone, where intermediate progenitors, outer radial glia, and migrating neuroblasts populate an expanding tissue layer, which will contribute to human neocortex. The corresponding neural tissue layers found in rodent developing cortex (middle) are depicted with analogous cell types and tissue layers labelled. Cortical organoid expanding neural cell layers include regions resembling the outer and inner subventricular zones termed ‘-like regions’ as they represent a model system rather than a fully functional characterized in vivo tissue. Illustration created with BioRender.com.

Beyond the macro-scale species differences, on the level closer to preclinical studies of neural cell biology, there exist several differences in cell development in the CNS. The neocortex germinal layer in murine outer radial glial cells populates a distinct neural layer in the developing cortex, the subventricular zone (SVZ) [50], whereas the expanded version of the outer subventricular zone (OSVZ) is unique to primates [51] and not present in rodents (Fig. 2) [52]. As an aside, human brain organoids have been shown to exhibit defined proliferative zones including an OSVZ-like region [53], although in general organoids grown in culture media lack a defined ventricular space with an ependymal border (Fig. 2). The OSVZ-like region in organoids has been identified from marker expression [53], confocal microscopy identifying characteristic mitotic behavior of outer radial glia (ORG), as well as single-cell transcriptome sequencing [54].

Expanding on biochemical and cellular differences between rodent model species and humans, the cell cycle is estimated to operate 2–3 times faster in mice than in humans [55, 56], with humans having a greater diversity of neural progenitor types, notably in the ventricular zone (VZ), humans have eleven distinct cell types compared with five cell types discovered in mice [57]. The neural circuitry is different in several ways; reviewed in depth by Matsui et al. [58], briefly cortical-striatal circuits involving the caudate nucleus and anterior putamen thought to be involved in executive function and social/language regions were not found to be conserved between mice and humans [59], and strong excitatory connections between single excitatory pyramidal cells and GABAergic interneurons are thought to be unique to the human neocortex [60]. The human brain is ~1000-fold larger than the mouse brain, with the greatest size discrepancy in the forebrain, although the midbrain has around tenfold more dopaminergic neurons in humans than in mice [57]. Beyond differences in volume and neural cell quantity, there are clear differences in brain folding from human gyrencephaly, as mice have wild-type lissencephaly, and while human doublecortin (DCX) mutations lead to lissencephaly phenotypes, there is no equivalent phenotypic difference of DCX mutations in mice [61].

On the level of neurotransmitters there similar exists several human-specific changes when compared with murine studies. Dopaminergic neurons contain substantially higher levels of dopamine in human neurons compared to murine neurons [62] as well as further limitations for direct comparison as reviewed previously [63]. This is notably relevant to psychiatry as levels of dopamine are highly correlated with psychiatric disease [64]. Other neuronal changes between human and mice are also readily apparent, the human neocortex contains significantly higher number and more diverse forms of gamma-aminobutyric acid (GABA) interneurons, whereas the density of dendritic spines are higher in mice [65].

The target of neurotransmitters, cell surface receptors, is another area of species’ difference, notably there are substantial differences in terms of the species-specific chemical neuroanatomy of 5-HT_2A_ receptors in the striatum [66]. Focusing on this receptor as the presently highly investigated receptor of ‘classic’ psychedelics in psychiatry, the 5-HT_2A_ receptors have species-dependent differences in dissociation kinetics with serotonergic psychedelics, with a prominent amino acid substitution in the orthosteric binding pocket [67].

When examining higher-level cortical electrical organization in species, there are noted differences including human hippocampal theta waves showing decreases in amplitude, frequency, and duration from rodent equivalents [68, 69]. As these species differences have been known for several years, an alternative methodology to study human neuroscience has developed out of growing neurons in the lab, an overview of which is discussed in the next section.

#### Overview of advancements in neuronal culture

Over one hundred and ten years have passed since Ross Granville Harrison made the advance of observing explanted neural explants from frogs in hanging drop culture, greatly propelling the ability to examine neurons ex vivo [70, 71]. In the more than a century since, molecular analytic techniques, microscopy, and a further wealth of advances have allowed researchers to visualize and characterize neural growth down to the nanoscale and make complex and nuanced discoveries.

The ability to differentiate neurons from fibroblast samples through induced pluripotency has particularly risen to prominence since the turn of the millennium and furthermore the expansions of cultures of cells into 3D have dramatically accelerated in recent years [17]. While reviewing efforts to create viable surrogates for studying the human CNS in labs, particular focus will be applied to the development of human induced pluripotent cells (hiPSCs) in tissue culture [72] and the evolution to more complex 3D culture methods from 2D planar cultures. 2D models will also be reviewed in some instances in further sections as they represent significant preliminary steps in understanding in vitro neuronal cultures, although ultimately lack reiteration of the complex cytoarchitecture and mechanotransduction found in living 3D tissue.

In the effort to expand in vitro cell cultures to 3D, non-planar hiPSC cultures have been in development since they were differentiated in the form of cortical neuro-epithelia in 2008. In likely the earliest report of brain organoids there were notable differences in the neuroepithelial structure between human and murine embryonic stem cell-derived cortical tissues, with a continuous versus polarized circular neuroepithelia, respectively, found after extended culture [73]. In 2012, hiPSCS grown in suspension with rostral neutralizing factors showed 3D structures, with transcriptome analysis indicating correlation with human cerebral cortical wall 8–10 weeks post-conception [74]. An unguided (non-patterned) differentiation study was introduced in 2013 where the organoids were derived from hiPSCs and showed distinct tissue layers [75]. Further advances in organoids have been diverse, following a theme of recapitulating wild-type functioning in a multitude of layers of biological complexity, from neurotransmitters to cell types and tissue organization.

As different research groups use individual protocols and have diverse discoveries, a common critique and noted limitation of current organoid technology has been batch-to-batch variability. This finding is improved by directed or guided differentiation toward specific brain regions, as opposed to unguided protocols, however, it remains an important consideration. Human cell-derived brain organoid models have been validated by multiple groups, and it has been demonstrated that reliable CNS tissue can be generated ex embryo in a reproducible and developmentally constrained manner that transcends individual organoids, experimental batch, genetic background, and sex [76]. Further validation efforts have shown that there was no inclination of gene expression toward disease-related genes in typical in vitro development and that there should not be a concern of transcriptional biases toward specific disease-related genes [77]. An additional study used organoids derived from postmortem fetal brain samples and compared the transcriptomes and epigenomes of isogenic fetal tissue, finding that the organoids matched closely with the human neocortex between 8 and 16 weeks post conception. Differentially expressed genes were noted particularly regarding the transition from stem cells to progenitors [78]. In essence, the overall epigenomic and the transcriptional program could closely mimic the developing human brain, however specific signaling pathways toward various cortical lineages remain essential, as unguided organoids do not demonstrate reliable reproducibility [22, 79, 80]. The recapitulation of in vivo cortical maturation has also been analyzed rigorously. In a pioneer study, 300 days old cortical organoids in vitro showed clear expression of postnatal protocadherin genes, suggesting that use of the Muotri lab protocol can achieve chromatin remodeling dynamics that mimic fetal-to-birth transition [81]. Using the same protocol, Trujillo et al. analyzed the cell populations and electrophysiological networks of 300 days cultured cortical organoids, showing neural oscillations comparable to preterm-to-birth EEG features [11]. At the molecular level, one study showed the equivalence of ‘early postnatal maturation’ is reached between 250 and 300 days in vitro, in terms of individual cell features such as NMDA receptor isoform switch. The authors note that their long-term culture of cortical organoids has non-trivial aspects, including a lack of survival of all cultures and an increase in differentiation variability after 400 days [82]. One research group has attempted to overcome the long-term survival barrier by using a sliced organoid culture system to aid in nutrient diffusion, leading to an expanded cortical plate with distinct cortical layers resembling third-trimester embryonic human neocortex [83].

Broadly, organoids technology has advanced in efforts toward understanding neural circuitry and neurotransmitters, notably pertinent to understanding psychiatry and psychotropic drugs. Organoids exhibit spontaneous Ca^2+^ transients in response to glutamate [84], and glutamate release can also be measurable by biosensors [85]. Functional synapses have been evident after 6 months in culture, and neurons have been shown to respond to stimuli, notably light stimulation [86], including with directed assembly of optic-vesicle-like structures [87].

To advance the complexity of neural systems modeled in vitro, several groups have published techniques where two or more brain regions are fused in a coculture system or in an assembly (“assembloids”). Techniques for combining 3D cultures of neural cells have been in development since at least 2013, where neurospheroids in millimeter-sized cubic molds were differentiated separately into hippocampal and cortical neuron cultures and then combined in coculture with their connections visualized [88]. Notable examples for coculture relevance in psychiatry include where dorsal and ventral forebrain organoids are fused, leading to GABAergic interneuron migration, which resembles cortical interneuron interaction between different brain regions [89], a pursuit of other forebrain models (considered the glutamatergic and GABAergic connection) [90, 91]. Other assemblies of in vitro cerebral organoids have been used to study complex cellular interactions [88], as well as circuit formation and maturation in longer-term cultures [92]. Another recent technique has sought to reconstitute the downstream connection of the CNS through the creation of neuromuscular organoids, which are composed of spinal cord neurons, Schwann cells, and skeletal muscle showing spontaneous contractile activity through a characterized neuromuscular junction [93]. Ultimately, while these models recreate an interaction of cellular migration and ingrowth between tissue types, they do not fully recapitulate the complex development of distinct tissues in the CNS.

Other advancements include adding astrocytes or microglia in coculture to help with synaptogenesis, leading to increased electrophysiological maturity over conventionally generated methods [94]. Further combination of cell types include addition of endothelial cells which have been combined with coculture media supplement with vascular endothelial growth factor in Matrigel^®^ coated organoids leading to primitive vascularization of the organoids in the form of cluster of differentiation 31 (CD31)—positive cells forming tubular channels [95]. Other techniques involved engraftment onto rodent models demonstrating infiltration of the organoid with host vasculature [96]. A functionally perfusable model of organoid vascularization in vitro has yet to be achieved, although it is an area of active research [97].

The development of inhibitory neurons in human 3D neural culture is another active area of research, and GABAergic mediation of glutamatergic activity is important for synchronized burst activity in older brain organoid cultures [98]. Organoids with electrical function have been shown to have advanced modeling characteristics, and calcium transients in the delta range show dysfunctional synchronicity with UBE3A suppression in a model of Angelman syndrome [99]. In another Angelman Syndrome model, the high synchronicity of spontaneous firing could be corrected by a novel compound, Paxilline [99]. Matured organoids have also demonstrated complex oscillatory networks analyzed with multi-electrode arrays (MEA), which can be compared to the developing EEG (Fig. 1) [11].

Technical engineering efforts in organoids have likewise developed as hiPSCs can be encapsulated in a bioink in a defined, scalable, and versatile platform and bioprinted in a 3D manner [100]. The 3D bioprinting approach has advanced to allow the printing of different cells in specific prearranged structures, developed into ‘miniaturized brains’ on a lab-scale use for specific conditions, including modeling glioblastoma multiforme and screening drug candidates [101]. Another technique involves organoids sliced and grown at the air-liquid interface showing outgrowing axons which demonstrated functional connectivity to mouse explants [102]. Stimulation paradigms have also been created to achieve controllable differentiation, including using systems with electrically active materials that have led to longer neurites through electrical stimulation [103]. Recently, “tissue-like” stretchable mesh nanoelectronics matching the mechanical properties of organoids have been integrated into organoids without interruption to development, enabling long-term stable recording of single-cell action potentials as well as complex oscillations initially reported in Trujillo et al. [11] offering a new look into early organoid electrical maturation [104].

### Modeling schizophrenia and psychosis

Schizophrenia is polygenic, hypothesized neurodevelopmental disorder, with a yet unknown molecular origin, although with a well-established theory of overactive dopaminergic signaling in the midbrain [64, 105]. Modeling schizophrenia presents a unique challenge, although emerging research is already providing valuable mechanistic insight with organoid models.

As schizophrenia is a disease of unknowns, there are a multitude of different paths to take toward mechanistic study, especially with in vitro experiments, and in the next section we will review some known features of schizophrenia and emerging discoveries with advanced cell modeling approaches. It has been suggested that there is a developmental or perhaps senescent cascade in schizophrenia as there is a mysterious gray and white matter reduction in schizophrenia patients [106, 107]. Along with this idea, diverse cell types in the brain have been thought to have abnormal development, especially microglia and interneurons. These changes have in common a downstream common pathway of altered dopamine signaling, which is generally treatment-responsive, albeit mostly in terms of ‘positive’ symptomatology, with antipsychotic therapy. This dopaminergic signaling is considered to be aberrant in several structures of the schizophrenic brain from the midbrain (mesolimbic) to the nigrostriatal (dorsal striatum) [108]. For an extensive review of schizophrenia modeling with hiPSCs with an emphasis on dopaminergic signaling please see the review by Collo et al. [109].

Schizophrenia has been modeled using hiPSCs since 2011, when idiopathic schizophrenic patient samples were used in 2D culture with neuronal phenotypes identified including diminished neuronal connectivity with decreased neurite numbers, postsynaptic density protein (PSD95)-protein levels (excitatory synapse marker), and decreased glutamate receptor expression. Further, there was altered expression in cAMP and Wnt signaling pathways and these phenotypes were rescued with added loxapine, an antipsychotic, to culture media [110]. Also, hiPSC lines were developed from defined DISC1 mutation patients in 2011 [111]. This mutation was discovered in a Scottish family through pedigree analysis in the Medical Research Council Cytogenetics Registry in 1990 [112], and established as a candidate gene for susceptibility to psychiatry illness in 2000 [113, 114]. Studies have continued with DISC1 hiPSCs in 2014 that showed transcriptional dysregulation of genes associated with synapses, and resultant defective synaptic vesicle release. Partially confirming this as a valid schizophrenia model, these phenotypes overlapped with prior studies from idiopathic schizophrenia patient-derived hiPSCs [115, 116]. It is worth noting that while patient specificity is a benefit in terms of isogenic disease modeling, in general due to the high cost of patient recruitment, sample isolation, and hiPSC generation, it remains difficult and often elusive to have sample sizes considered broadly representative of disease populations.

Moving forward with the relevance of DISC1 to schizophrenia modeling, previous studies have shown that both DISC1 and NudE Neurodevelopment Protein 1 (NDEL1) can regulate the cell cycle by interacting with glycogen synthase kinase 3 beta [117] and LIS1-dynein [118], respectively. Research has also shown DISC1 and NDEL1 interact and regulate the cell cycle and can govern cortical size [119, 120]. Human forebrain organoids derived from patient hiPSCs carrying a DISC1 mutation were demonstrated to affect neural development through dysregulation of radial glial cell proliferation, as the neural stem cell population is controlled through DISC1 and NDEL1 during human forebrain development [121].

As discussed, an area of active research in schizophrenia is altered neurodevelopment. A further finding in schizophrenia patient-derived organoids was disruption of the developmental strata, including increased Ki67+ neural progenitor cells (NPCs) in the intermediate zone and atypically placed deep subcortical neurons, accompanied by significant loss of T-box, brain 1 (TBR1) positive pioneer neurons from the top cortical layer. Overall abnormal subcortical neurogenesis was consistent with transcriptome studies of NPCs from schizophrenia hiPSCs. Also apparent was visibly diminished different pan-neuronal expressing fibers and random directionality of calretinin-positive neurites [122]. Glutamatergic pyramidal neurons and GABAergic interneurons, of which calretinin-expressing is a subtype, have been implicated in the development of schizophrenia in humans [123]. This study overall suggests interneuron directionality may disrupt cortical connections and suggests a nuclear fibroblast growth factor receptor 1 dysregulation on neuro-ontologic gene programming [122].

Further investigating the exhibitory and inhibitory developmental cascade, glutamatergic and GABAergic neurons in independently differentiated forebrain subdomains were then combined in coculture recapitulating the saltatory migration of interneurons. To develop this assembly these sub-brain regions modeled as human cortical spheroids and human subpallium spheroids were placed in a conical tube and fused together after 3 days. This migration was reminiscent of interneuron migration in the fetal forebrain [91]. Given the critical role of cortical interneurons in preserving excitatory-inhibitory E/I balance and its implication in schizophrenia, a long-sought goal is to recapitulate the human medial ganglionic eminence (MGE), which is where parvalbumin interneurons are primarily generated. There are many challenges to generating pure MGE-derived GABAergic cells, notably difficulties fine-tuning protocols, obtaining the necessary in vitro environment, and maintaining cells for a long enough duration for maturation [124–126]. Several techniques have been developed, including spinner culture that produces homogeneous human cortical inhibitory cells without feeder cell coculture, as feeder cells may introduce impurities [127]. Despite progress, it is clear that while hiPSC-derived interneurons capture developmental stages prior to mid-gestation, they may incompletely represent the full diversity of cell subtypes [128]. The MGE has also been recapitulated in the context of human interneuron migration and integration, with the fusion of transcriptionally defined MGE and cortex-specific organoids [129]. Importantly, human-specific cortically born interneurons [130] might have a more prominent contribution to schizophrenia, even contributing to the alterations in gamma oscillations observed in patients [131, 132]. Thus, it would be important to dissect the contribution of the different types of interneurons to disease pathology.

Additional efforts to determine higher-level physical brain phenotype changes in schizophrenia return to previous studies of DISC1. The group involved had previously made an isogenic hiPSC model which showed in 2D culture that the balanced translocation of a loss of long DISC1 isoforms had a reduction in the production of T-box brain protein 2 (TBR2)—positive NPCs and an increase in baseline Wnt signaling [133]. The group expanded on this work by creating 3D cerebral organoids with DISC1 disruption (DISC1 exon 8 disruption), which were found to be morphologically distinct from controls. These DISC1 disrupted organoids had smaller, disordered neural rosettes, which could be rescued with a Wnt agonist. These phenotypes were not thought to be able to be studied in monolayer cultures, thus necessitating 3D cultures which demonstrate 3D cytoarchitectural changes.

Whether or not DISC1 leads to clinically significant brain phenotypic changes is controversial as patients with DISC1 disruption do not have gross cortical structural defects, thus the phenotype may be amplified due to inherent qualities of the organoid system, including lack of support systems (microglia and neurovasculature). However, there are animal models with a reduction in cortical volume, enlarged ventricles, and thinning of the cortex with DISC1 disruption [134–138]. In addition, in brain imaging studies looking at subjects with DISC1 mutant alleles, it was found white matter thickness was significantly reduced, a finding shared with schizophrenia patients [139–141]. The global brain development transcription factor (BRN2) levels were also decreased in the organoid study, and transcription factor GS Homebox 1, (GSX1) which patterns ventral-telencephalic patterning was also affected as well as markers related to interneuron development, which as discussed is related to schizophrenia and other mental illness [140, 142]. Convergently DISC1 is necessary for the migration of cortical interneurons (in a mouse model) [143].

Critical to the modeling of downstream dopamine dysregulation in schizophrenia, a human midbrain dopaminergic system was developed which optimized tyrosine-hydroxylase expressing dopaminergic neurons. It was also shown that toxin-induced dopaminergic neuronal cell death was preserved in midbrain organoid models. The authors note this could replace the ‘highly used and ethically compromised’ 6OH-Da rodent model, with ‘great potential for drug screening’ [144].

Further research questions on schizophrenia regard the contribution of neuroglia to pathophysiology. There is a significant increase in microglia density in patients with schizophrenia as compared with matched controls, and it is unknown if this is an environmental or genetic component as stress leads to elevated microglial activity in hippocampal regions [145]. Other findings in schizophrenia include significantly increased microglia in white matter [146] and decreased free-water corrected fractional anisotropy in white matter tracts [147]. Schizophrenia patients can also exhibit a significant effect toward increased expression of proinflammatory genes [148]. Along with the increased volume of the lateral ventricles, and general brain volume loss, this is one of the most consistently observed brain alterations in schizophrenia [149].

Microglia are centrally acting neural immune cells with ‘trained innate immunity’ with research leading to a possible role in schizophrenia [150]. To model the microglial contribution in 3D cultures, hiPSC-derived macrophages and microglia have been cocultured in 3D platforms [151]. Organoids transplanted with primary microglia demonstrated a role of microglia in neural circuit development, with an increase in network-level synchronized activity in organoids that contained transplanted microglia [152]. Synaptic material was discovered in microglia cells which may have led to more efficient pruning [152].

Further work has used schizophrenia patient-derived organoids to identify differences from controls in progenitor and differentiated neurons, discovering two novel gene growth and transcription factors, Pleiotrophin and BRN2, respectively. These factors were downregulated at the mRNA level to the peptide level within progenitors and rescue experiments could restore reduced neuron number in schizophrenia patient-derived organoids [153]. Interestingly the same work reveals ‘an intrinsic enrichment’ for interferon-induced transmembrane factor (IFITM3), which is enriched in postmortem schizophrenia brain and may reflect increased neuroinflammation, and oxidative stress. IFITM3 also may mediate perinatal immune activation effects and has been discussed as a novel drug target for schizophrenia [153, 154].

In a recent study, the bench to bedside approach was utilized as hiPSC-derived neurons were differentiated from fibroblasts in schizophrenia patients and controls, and electrical activity compared to patient performance on a variety of cognitive tasks. It was found that schizophrenia patient-derived neurons had altered Na^+^ channel function and increased frequency of GABA transmission. Glutamate transmission appeared unchanged between groups and the phenotype is suggested to reflect an alteration in synaptic E/I balance. The cognitive tests of schizophrenia patients correlated to spontaneous excitatory postsynaptic transmission amplitude, and the number of Na^+^ peaks showed association with positive symptoms of schizophrenia [155].

The question of heritability in schizophrenia as touched on previously is curious as twin concordance is estimated to be 50% [156]. Patient-derived human cerebral organoids from discordant twin pairs for schizophrenia showed enhanced GABAergic specification of patient NPCs, further demonstrating ‘unbalanced’ specification of E/I balance may underly a common pathway of psychoses [157]. While evidence of mechanistic etiology in schizophrenia found with advanced organoid modeling may appear diverse and somewhat disparate, given schizophrenia likely has a final common pathway leading to dopamine dysfunction, it is worthy to pursue various avenues toward understanding contributors. In particular, one theory suggests that oxidative stress leading to interneuron impairment in neurodevelopment is a consequence of various upstream perturbations. Further incorporating the ‘two-hit’ model would mean a sub-threshold immune challenge around adolescence primes microglia activation, causing reactive oxygen species production and specific convergent parvalbumin/perineuronal net dysfunction conserved across psychosis models both of animal and in vitro origin [158]. Considering in vitro models successfully examine disease responses of inflammation/microglia contributions to neurodevelopment and E/I balance, with an initial suggestion of correlation with patient-centered cognitive outcomes, this would suggest these models have a valuable contribution to convergent disease understanding.

### Modeling neurotransmission and mood disorders

Despite decades of research, most psychiatric disorders remain without consensus etiologies, however, they are generally theorized to be dependent on the action of neurotransmitters. The diverse receptor interactions of known psychotropic medications indicate that this is a clinically relevant hypothesis. While most of the organoid literature focuses on glutamatergic/GABAergic signaling, organoids have been developed to replicate dopaminergic and serotonergic signaling and have led to valuable insights into the mechanism and pharmacokinetics of antidepressants. In this section, we will explore human cell-based models which have shown promise in elucidating the origin of mood states and effects of clinical treatment.

In one study of well-characterized MDD patients, hiPSCs were generated from selective serotonin reuptake inhibitor (SSRI)-remitters and SSRI-non-remitters, and serotonergic neurotransmission was examined in differentiated forebrain neurons. It was found that non-remitter patient-derived neurons displayed serotonin-induced hyperactivity downstream of upregulated serotonergic receptors, not seen in healthy or remitter samples. The authors indicated postsynaptic forebrain hyperactivity downstream of SSRI treatment could play a role in treatment resistance [159].

In a demonstration of the advantage of advanced engineering of organoids, human choroid plexus organoids that created cysts of CSF-like fluid were used to test transport across the internal fluid of the organoids. Bupropion was found to cross into the organoid internal fluid and reached baseline levels similar to what is found in vivo [160].

Another study used a class of antidepressants known as monoamine oxidase (MAO) inhibitors (MAOI). The study used tranylcypromine (2-CPA, Parnate®), a nonselective and irreversible MAOI, and tested the effect of exposure of 0–10 μM 2-CPA on human cerebral organoids. They found that 2-CPA impaired neural growth with a noted decrease in expression of Ki-67, and a cleaved caspase 3 expression increase. Global decrease of lysine-specific demethylase 1a (LSD1) and an increase in histone H3 lysine K4 (H3K4) methylation was also observed. Intriguingly LSD1 has sequence homology with MAO [161], and the authors note the next step into finding a mechanism of neurotoxicity in the MAOI will be to determine a direct link with LSD1 histone demethylation inhibition and tranylcypromine induced neurotoxicity [162].

Differentiated neurons have been studied for a variety of mood disorders, including bipolar disorder, an important mechanistic distinction from unipolar depression as a bipolar diathesis indicates an improved response to mood stabilizers relative to antidepressants [163]. In one study hiPSCs differentiated from bipolar patients showed differential expression of genes involved in calcium signaling, which was also shown to be sensitive to lithium pre-treatment of the bipolar patient-derived cultures. The study also found that bipolar patient-derived neurons may have been closer to ventral neuronal subtypes, and proposed lithium as a mechanism that would activate Wnt pathway signaling and ‘dorsalize’ early CNS progenitors [164].

In further bipolar disease research in a 2D hiPSC model, hippocampal dentate gyrus-like neurons derived from bipolar patients detected mitochondrial abnormalities as compared to controls. In addition, using patch clamp and somatic Ca^2+^ imaging, hyperactive action potential firing found in hiPSCs of patients with bipolar disorder was selectively reversed with lithium, although only in patients who were classified as lithium responders [165].

In a brain organoid model with cells derived from bipolar disorder patients from a research group that published a similar methodology with schizophrenia patients [166], genome-wide association studies revealed ‘downregulation of genes in cell adhesion, neurodevelopment, and synaptic biology,’ significantly locating a ‘central hub’ of neurocan (NCAN). Ontology analyses also pointed to deficits in the endoplasmic reticulum and in MEA analysis, which showed decreased response to stimulation and depolarization [167].

### Environmental and substance abuse, acquired cognitive disorders

As psychiatrists treat all patients with behavioral disturbance, the etiologies are not limited to mood and psychotic disorders thought to be polygenic or neurodevelopmental in origin. Psychiatrists also treat individuals who suffer from substance use disorders, as well as cognitive disorders, including from exposure to environmental insults, notably prenatal exposure to alcohol and other substances. These disorders incur a high cost to the individual and society, and several labs have developed techniques for the in vitro modeling of pathogenesis and treatment of these conditions, demonstrating the utility of 3D cell culture techniques for propagating insight into environmentally-induced disorders.

#### Autism spectrum disorder

Autism studies are discussed in depth in other reviews concerning 3D in vitro models of neurodevelopmental disorders [22, 71, 168–176]. Here, we will focus on the utility of human stem cell models for non-syndromic idiopathic ASD, which comprises roughly 70% of cases. In general, it is thought that genes associated with ASD converge on pathways of chromatin remodeling, neurogenesis, cortical lamination, and neuronal and synaptic maintenance [170]. Initial studies on idiopathic autism used hiPSC to model genetic variants of unknown significance, showing the contribution of this approach to validating rare mutations [177]. An alternative approach has been to use cells differentiated from hiPSC from individuals with severe idiopathic ASD and microencephaly as endophenotypes, where transcriptome and gene network analyses showed upregulation of genes in cell proliferation, neuronal differentiation, and synaptic assembly [178]. Brain organoids from similar cohorts also exhibited an accelerated cell cycle and overproduction of GABAergic inhibitor neurons, thought to be caused by FOXG1 [179]. More recent work with ASD subject derived-neurons showed ASD neurons with aberrantly complex neurite structures, and also an increased thickness of the cortical plate [180]. A non-cell-autonomous contribution of astrocytes to idiopathic autism has also been revealed [181]. A further recent study using idiopathic ASD-derived brain organoids found that aberrant calcium signaling in astrocytes can affect neuronal activity and even disrupt behavior upon transplantation in mice [182]. Similar to schizophrenia, the work with idiopathic ASD suffers from comparable challenges, as this patient population is quite heterogenous from a genetic perspective. Nonetheless, these pioneer works are suggesting that common molecular and cellular pathways can be found, providing fertile soil for future interventions and better treatments.

#### Alcohol exposure to organoids

Brain organoids that replicated first-trimester development were exposed to 50 mM of ethanol, which led to premature neural differentiation, more pronounced with prolonged exposure. This ‘hyperdifferentiation’ of glutamatergic neurons with conserved differentiation of GABAergic neurons was speculated to change E/I balance. Other changes included expression changes of neurogenesis-related genes in the Hippo pathway, a pathway which regulates brain size, notably including roof plate-specific spondin-2 (RSPO2), known to be involved in craniofacial morphogenesis. Cell death was also induced by ethanol exposure [183].

In another model system, a novel alginate hollow fiber system for preparing brain organoids was used [184], and organoid exposure to 50 mM ethanol in culture media again induced premature neuronal differentiation. The study also identified putative new genes identified with ethanol exposure, notably, biological pathways associated with long-term depression were also correlated [183].

#### Nicotine, stimulant, and psychoactive compound exposure

The developmental consequences of prenatal nicotine exposure is an evolving and complex topic. However, there is a demonstrated association between prenatal nicotine exposure and altered brain structure and function as well a correlation with behavioral issues, notably ADHD, in exposed offspring [185]. Efforts have been made to model prenatal nicotine exposure in organoids, and it was found nicotine addition to culture media disrupted brain regionalization and cortical development with abnormal neuronal differentiation and migration [186]. Another approach used embryoid bodies, which were differentiated and organized in brain organoids under continuous perfusion with 1 uM or 10 uM nicotine. This approach used an intriguing combination of 3D culture with a lab-on-a-chip enabling precise control of intoxicant concentration to study cytoarchitectural neurodevelopmental changes [187].

Prenatal cocaine exposure is another concerning neurodevelopmental insult, also studied in a 3D organoid model. In the model, it was found that cytochrome P450 3A5 (CYP3A5) mediated the generation of reactive oxygen species with cocaine exposure, as well as inhibition of neocortical progenitor cell proliferation, induction of premature neuronal differentiation, and interruption of neuronal tissue development. Knockdown of CP3A5 reversed these findings, suggesting CYP3A5 as a target for therapeutics designed to rescue harmful neurodevelopmental effects of in utero cocaine exposure [188]. Similar approaches have been used to study prenatal cannabis exposure showing cannabis exposure reduced neuronal maturation, downregulated cannabinoid type 1 (CB1) receptors, and impaired neurite outgrowth [189]. In an additional study, prenatal methamphetamine exposure in organoids suggested changes in neuroinflammatory gene expression [190].

Another area of research into psychoactive substances concerns 5-methoxy-N,N-dimethyltryptamine (5-MeO DMT), an ‘entheogenic serotonin-like molecule,’ and structural analog of serotonin and melatonin. This substance is reportedly associated with cognitive gain and antidepressant effects. In studies using human cerebral organoid models, anti-inflammatory effects were found through downregulation through toll-like receptor and 5-HT_2A_, 5-HT_2C_, downregulating nuclear factors of activated T-cells (NFAT) and kappa B (NF- _Κ_B). Treated organoids exhibited downregulation of metabotropic glutamate receptor 5 (mGLuR5), which has a role in several drugs of abuse; notably mice lacking mGLuR5 do not self-administer cocaine. 5-MeO-DMT also caused significant upregulation of ephrin-B2 (EFNB2), and ephrin type-B receptor (EPHB), and other secondary messengers in dendritic spine formation [191].

#### Neurotoxic and neuroprotective molecules

In experiments not limited to exogenous psychoactive substances, numerous studies have sought to determine how risk factors in the supportive developmental environment may lead to downstream consequences affecting nervous system functioning. In this section, we will review a selection of other factors relevant to psychiatric disease and cognition, from toxic to protective molecules, trace elements, and hypoxic conditions.

Vincristine is a chemotherapeutic with the potential for a high degree of neurotoxicity, albeit with a controversial mechanism for damage to exposed neural tissue [192]. In an organoid model, 48 h treatment of vincristine showed a reduction of neurons and astrocyte numbers with dose dependency. Vincristine was also shown to impair key cytoskeletal proteins tubulin and fibronectin [193]. This model demonstrates a modality for determining novel insights into medication with neurotoxic properties and, more broadly, a demonstration of effective preclinical screening modalities.

A contrasting utility of organoid models was shown through testing human brain organoids with minocycline, a tetracycline antibiotic, which is also under investigation for treatment of early psychosis due to its theorized neuroprotective qualities, and its high degree of lipophilicity and thus ability to cross the blood-brain barrier. This compound was tested in a model of neonatal hypoxia, and morphological atrophic changes and hypoxic stress gene expression changes were rescued by minocycline in this model [194].

#### Hypoxia

Hypoxia as a condition is highly relevant to preclinical disease testing, as prenatal hypoxia correlates with postnatal morphological changes in brain structures involved in learning and memory and impaired development of cognitive functions [195]. In one experiment, human brain organoids exposed to 48 h of <15% O_2_ modeled mid-gestation human cortex in conditions of hypoxic encephalopathy of prematurity and second-trimester placental insufficiency. Several notable changes were found, notably TBR2-positive progenitors in the SVZ were particularly affected by oxygen deprivation. This response was thought to involve the unfolded protein response (UPR), a response pathway to endoplasmic reticulum stress. It was found that the UPR modulator, integrated stress response inhibitor, (ISRIB) which is BBB permeable, prevented the hypoxia-related TBR2 defects [196].

In an additional work, organoids were cultured in a hypoxic chamber at 3% O_2_ for 24 h, and 28 days after differentiation, and it was found that the progenitor population of ORG had immediate and prolonged apoptosis. As the ORG are more prominent specifically in primates, (Fig. 2) this paradigm showed human specific progenitor changes with novel insights into the etiology of cortical dysgenesis associated with hypoxic insults [197].

#### Trace elements

Other miscellaneous relevant developmental factors include specific micronutrients or trace elements, as little is known about the in vivo developmental composition of the developing human CNS, although metal metabolism is known to be disrupted in neurodegenerative and neuropsychiatric disorders. Studies detailing elemental composition in organoids showed distinct levels of phosphorus, potassium and sulfur. It was also shown that trace elements change distribution inside organoids during differentiation. Potassium was seen to be first concentrating at the periphery then became evenly distributed as differentiation continued. Zn was more homogenous and then became peripheral at 45 days. Glutamate occupied the outer portion of organoids, interesting because Zn is present at high levels in glutamatergic synapses and Zn dependent metalloproteinases facilitate neural migration and outgrowth. These metal concentrations are clinically relevant as developmental deficiencies in Zn nutrition are associated with memory defects and maternal Fe deficiency may be a risk for schizophrenia in offspring [198, 199].

#### Future directions

Building on over a century of lab culture of neural tissue, human brain organoid models have increased in complexity in phenotypes and relevance to human development and circuitry, particularly in the past decade. Although these models do not have the benefit of behavioral outputs found in animal models, they are increasingly relevant to psychiatric diseases of uncertain etiology, whether polygenic, neurodevelopment, or acquired. Moreover, organoid models are likely to be increasingly relevant to translational researchers, including in searching for novel therapeutic compounds. Disease models will similarly be useful for psychiatrists seeking to further understand the complex neurochemistry of commonly treated diseases as they portend to known kinetic actions of neurotransmitters and upstream contributions of neurodevelopmental and environmental interactions. Areas of ongoing improvement include, but are not limited to, increasing recapitulation of the complex interaction between developmental regions of the CNS, and further maturation and sophisticated analysis of electrical signals, including those produced from stimuli.

Human organoid studies nevertheless face several limitations, related to many features including indistinct batch reliability, and limited recapitulation toward maturing in utero development. Limited batch reproducibility in unguided organoids necessitates directed patterning to increase subtype specificity, and while assemblies of multiple organoids can recreate aspects of CNS subtype interactions, they remain limited in reiterating the full scope of CNS development. Perfusable vasculature also remains to be solved with in vitro models and the BBB remains a difficult to recapitulate phenomenon. As these technologies develop, it will also be important to increase the ability to record efferent responses to stimuli, as well as increase the ability for sensory input, although these will necessitate a continued discussion of best practice ethical principles.
